# Study of Patients’ Willingness to Pay for a Cure of Chronic Obstructive Pulmonary Disease in Taiwan

**DOI:** 10.3390/ijerph13030273

**Published:** 2016-03-01

**Authors:** Yi-Ting Chen, Yung-Hsiang Ying, Koyin Chang, Ya-Hui Hsieh

**Affiliations:** 1 Hualien Buddhist Tzu-Chi General Hospital, Hualien City 970, Taiwan; yitingchen@tzuchi.com.tw; 2 Department of Business Administration, National Taiwan Normal University, Taipei City 106, Taiwan; 3 Department of Healthcare Information and Management, Ming Chuan University; Taipei City 111, Taiwan; hsieh_yahui@hotmail.com

**Keywords:** chronic obstructive pulmonary disease (COPD), health-related quality of life (HRQL), comorbidities, willingness to pay (WTP), disease severity

## Abstract

*Objectives*: Chronic Obstructive Pulmonary Disease (COPD) is one of the fastest growing causes of death worldwide. However, few studies, if any, have been conducted that have investigated patient profiles in Asia. This paper analyzes patient willingness to pay (WTP) as a function of patient disease severity, health-related quality of life (HRQL), and smoking behavior in Taiwan. *Study Design*: A cross-sectional survey was conducted using in-person interviews with COPD patients. A hypothetical scenario was designed and presented to ascertain each subject’s willingness to pay (WTP) for a cure for COPD. *Methods*: A survey of subjects with COPD was performed in Taiwan. The contingent valuation method (CVM) was employed to measure patient financial burden, which was analyzed along with covariates that included various types of health-related quality of life (HRQL), severity level, and demographic background. Multivariate regression and simulation methods were employed for analysis. *Results*: A total of 142 subjects were interviewed, with an average annual WTP of approximately $1422 USD (or 42,662.37 NTD, New Taiwan Dollars). The annual WTP for patients 55 years of age or younger, $5709.06, was the highest and equivalent to approximately one-third of Taiwan average annual personal income or quadruple the spending amount of the Taiwan National Bureau of Health Insurance (NBHI) for each COPD patient. Current cigarette smokers were willing to pay a substantially higher amount than former smokers and nonsmokers, which reflects a psychological desire for redemption in COPD patients. *Conclusions*: The results of this study provide directions for the relevant authorities regarding the alleviation of suffering as a result of COPD. Appropriate health promotion measures, such as measures to reduce tobacco usage, early diagnosis, and active treatment, may be necessary to contain the escalating costs related to COPD and to prevent this epidemic from worsening.

## 1. Introduction

Chronic obstructive pulmonary disease (COPD) is a treatable and preventable disease with fast increasing morbidity and mortality around the world. COPD has been one of the most significant long-term conditions that face society [1] and is currently the fourth leading cause of death in the United States (U.S.). It will become the third ranked major disease in the world by 2020 [2,3]. In Taiwan, COPD has been the ninth leading cause of death for the previous decade and is one of the fastest growing diseases, with an annual patient growth rate of approximately 9.33%. The prevalence rate of COPD is approximately 4%–5% for the population 40 years and older [4] Studies indicate that COPD is almost always caused by smoking, which accounts for as many as 9 of 10 COPD-related deaths [5,6]; it is also directly associated with lung cancer, one of the leading causes of death in Taiwan. Although smokers comprise only an estimated 21.74% of the population in Taiwan, which is slightly higher than the U.S. percentage (18.1%) [7] but lower than the percentage in most developed countries, the high incident rate of the disease is strongly associated with smoking and thus requires healthcare authority attention. The burden to the COPD patient is high in terms of both health-related quality of life (HRQL) and health status. Patients suffer from poor physical conditions and distressing symptoms that require frequent hospital visits/admissions as the disease worsens. Because COPD is chronic and patients typically do not sense the seriousness of the disease at its early stage, they sometimes wait too long before seeking proper treatment, which causes substantially higher medical expenses than if they received treatment earlier. Studies regarding the financial burden of COPD abound. In the U.S., the annual expense that results from COPD ranges from $2003 to $43,461 per patient [8,9], which translates to a total expense of $29.5 billion in direct health care expenditures, with additional costs not captured in claims because of under-diagnoses and misdiagnoses [10,11]. In other countries, COPD costs the Canadian healthcare system as much as $1.5 billion per year [12]; it costs $9.9 million annually in Singapore [13], 1.5 billion Euro annually in the UK [14], and an estimated €38.7 billion ($51.2 billion USD) annually in Europe with approximately 60% of the total costs of COPD attributable to indirect costs, such as workdays lost, disability pensions, and lost tax revenues because of COPD [15,16]. The Taiwan National Health Insurance system has spent approximately $27 billion NTD ($900 million USD) annually for the treatment of over 700,000 COPD patients [17]. 

Most modern medical research on the disease has focused on the prolongation of life in patients with COPD. The management of COPD patients in the future, however, will gravitate toward improving quality of life (QOL) in addition to survival. Studies have been conducted to evaluate patient HRQL and have demonstrated that poor symptom control and exacerbations can lead to limitations in functioning and impaired HRQL [18,19,20]. Although a vast number of COPD studies exist, few studies, if any, have simultaneously conducted comprehensive research regarding patient HRQL, comorbidities, and cost aspects. Thus, the purpose of this study was to investigate the willingness to pay (WTP) for a cure of the disease in patients with COPD in Taiwan while controlling for factors such as HRQL, severity level, and smoking pattern. These findings will enable us to gain a better understanding of the suffering of COPD patients and hopefully arouse more attention from the public and increase efforts by government authorities to alleviate patient burdens.

## 2. Methodology

### 2.1. Study Design

A cross-sectional survey was conducted using in-person interviews with COPD patients. This survey was conducted from January 2013 through July 2014 using a specially designed questionnaire that adopted a retrospective, patient-based point of view and was developed in four phases. 

### 2.2. Study Population

Two hundred forty-eight patients were invited to be interviewed from two medical centers in northern and eastern Taiwan. Only outpatients were surveyed because patients with COPD are treated primarily through outpatient services in Taiwan unless they are end-stage ill patients with serious complications. Thus, extreme cases were excluded to avoid an upward bias of the WTP result. Patient recruitment was initiated by the pulmonologists at the hospitals. Eligible patients were referred to specially trained interviewers who provided the patients with complete information regarding the study and obtained consent from individuals who agreed to participate. The inclusion criteria of the subjects were as follows: (i) 40 years of age or older; (ii) registered with a pulmonary clinic under a COPD diagnosis, which was defined by an FEV1-to-FVC ratio (the ratio of FEV1 (forced expiratory volume that is exhaled at the end of the first second of forced expiration) to FVC (forced vital capacity)) of less than 0.7 as measured 20 min after the administration of albuterol; (iii) in a clinically stable condition and receiving appropriate therapy; (iv) Chinese/Taiwanese speaking; and (v) having the ability to provide informed consent. The exclusion criteria were as follows: having an illness other than COPD that was likely to result in death within one year and asthma, which was defined as an increase in FEV1 of more than 15% or 200 mL above the baseline value after the administration of a bronchodilator.

### 2.3. Development of Questionnaire

First, the contingent valuation mention (CVM) was consulted to determine the general structure of the instrument. Three clinical experts subsequently reviewed the instrument for content validity. To deduce the proper amount of the patients’ WTP, an open-ended question was surveyed in 30 patients to determine the first round of WTP bids. Finally, the instrument was pilot-tested in 10 patients to assess its clarity. The survey instrument consisted of 6 sections: the patient’s medical history, demographic questions, medical utilization, HRQL, clinical information, and WTP for a cure.

### 2.4. Main Outcome

The main outcome of this study was the WTP for a COPD cure, which indicates a patient’s suffering from the disease and is one of the most well-established approaches for estimating disease costs, including direct, indirect, and intangible costs (in some cases, the EQ-5D Index can be negative if the QOL is worse than death. The translation formula can be obtained from the EuroQoL Group Monograph series published by Springer.). A higher WTP implies that a patient suffers from a more serious disease condition, which, in turn, causes greater spending on treatments and other spending towards relieving pain and emotional setbacks. This approach has been applied to many diseases, such as diabetes, various cancers, dermatology, reflux disease, and rheumatoid arthritis. This paper investigated the determinants of WTP and assessed the extent to which each determinant contributed to the variation in WTP. Thus, we can gain practical knowledge regarding patients’ general conditions and how these conditions are associated with patients’ suffering measured by the WTP. We compared the regression coefficients of the determinants, with those determinants that lower the WTP implying a positive impact on the disease condition.

The WTP of each subject was assessed following the procedure of Lang, Chang, and Ying (2012) [21]. Five groups of bids were designed based on the pilot-test results from the 30 individuals who were included in the previously described open-ended question survey, which inquired about the WTP for a cure for COPD. Other questions, such as those involving demographic data, were not included in the pilot questionnaire. The initial bid of WTP, which ranged from $5000 NT to $25,000 NT in $5000 intervals, was divided into 5 categories centered by a round figure near the mean value of the pilot test. The second-round bids were subsequently created by adding or subtracting the standard deviation of the first bid value (rounded to $3000 NT in the study) depending on whether the answer to the first round was yes or no. Each respondent was randomly assigned to one of the five groups. Because the BNHI has provided largely comprehensive treatments for COPD, the WTP amount obtained from each subject implied that the patient desired additional coverage.

### 2.5. Covariables

COPD is a chronic disease. Patients with COPD are associated with a higher rate of comorbidities than are patients without it. The presence of different comorbidities in patients may affect his/her WTP. Therefore, it is of interest to investigate how individuals with COPD suffer from these complications or other related health problems. In the medical history section of the questionnaire, questions on the existence of comorbidities were included. For the demographic data, questions on age, education, income, marital status, and employment were included. According to the Global Initiative for Chronic Obstructive Lung Disease (GOLD), COPD severity is measured by a combination of impacts including spirometry, CAT (COPD assessment test), CCQ (Clinical COPD Questionnaire), and mMRC (the modified British Medical Research Council) scale [22]. In the present study, both spirometry and CAT were employed as the control variables for severity level. For HRQL, the WHOQOL, EQ-5D, and SF-36 measures were employed in the questionnaire as quality of life indicators. 

The concept of HRQL was developed to include measures of the impact of disease and impairment on daily activities and behaviors (e.g., Sickness Impact Profile [23]), perceived health measures [24], and disability/functional status measures [25]. The management of COPD is largely symptomatic; thus, patient-reported outcomes (PROs) that evaluate HRQL are important to evaluate the treatment and management of COPD. Disease-specific measures can provide insight into specific aspects of HRQL, whereas generic HRQL measures have the advantage of allowing comparisons across different patient populations; however, the latter measures may be less sensitive to changes in HRQL compared with disease-specific measures [26]. This study used the World Health Organization QOL (WHOQOL) BREF, EQ-5D, and short-form health survey with 36 questions (SF-36) to evaluate patient HRQL. 

#### 2.5.1. WHOQOL

The WHOQOL-BREF assessment was developed by the WHOQOL group. The present study used the short form of the WHOQOL-100, the WHOQOL-BREF, which contains 26 questions; these questions are structured in four domains, including physical health, psychological condition, social relationships, and environment. The results of these survey questions were transformed into scores that ranged from 0 to 100 for each of the four domains. The details of the questionnaire can be found in the instruction manual on the WHO website [27].

#### 2.5.2. EQ-5D

The EQ-5D questionnaire is a standardized measure of health status developed by the EuroQoL Group; it has demonstrated its usefulness in major therapeutic areas [28] and health surveys across various general European populations [29,30]. Five dimensions of an individual’s condition are targeted: mobility, self-care, usual activities, pain/discomfort, and anxiety/depression. The response results were translated into an index (the EQ-5D Index) that ranged from 0 (death) to 1 (perfect health) for subsequent analyses. Because there is no specific value set of utility weights for the Taiwan population, this study adopted the most robust valuation set, the UK TTO set, as suggested by the EuroQoL Group.

#### 2.5.3. SF-36

The original SF-36 originated from the Medical Outcome Study (MOS), which was conducted by the RAND Corporation. It is a multi-purpose, short-form health survey with 36 questions. Two psychometrically based measures were recorded: the physical and mental health component summary measures (PCS and MCS). These scores range from 0 to 100, with higher scores indicating a better self-reported health status. The general population has PCS and MCS scores close to 50. Scores higher or lower than 50 indicate a better or worse health status than the general population, respectively. 

Both the EQ-5D and SF-36 have proven useful in surveys of general and specific populations to compare the relative burden of diseases and to differentiate the health benefits produced by a wide range of different treatments. Recently, both measures were judged to be the most widely used generic patient-assessed health outcome measures [31]. The inclusion of these two QOL measures is appropriate for investigating patient HRQL; additionally, further comparisons across diseases can be achieved with these measures. 

#### 2.5.4. Spirometry

The spirometric classification of airflow limitation, *i.e.*, forced expiratory volume in 1 s (FEV1), has long been used for assessing and diagnosing COPD [22]. The present study included spirometry as one of the measures of severity. Four grades are classified:

1. Mild: FEV1 ≥ 80% predicted
2. Moderate: 50% ≤ FEV1 < 80%
3. Severe: 30% ≤ FEV1 < 50%
4. Very Severe: FEV1 < 30% predicted

#### 2.5.5. COPD Assessment Test (CAT)

In addition to spirometry, a few other severity measures are recommended by the Global Initiative for Chronic Obstructive Lung Disease (GOLD), including the CAT [22,32,33]. The CAT was developed to be applicable worldwide, and validated translations are available in several languages. Its results correlate very closely with health status as measured using the St. George Respiratory Questionnaire (SGRQ), and it is regarded as both reliable and responsive [34]. The CAT scores range from 0 to 40; greater CAT values imply more severe symptoms or a greater impact on patients.

### 2.6. Statistical Methods—Contingent Valuation Method (CVM)

To assess disease costs, this study employed a double-bounded dichotomy CVM for the statistical analysis. The CVM is the most widely used method to evaluate non-measurable economic benefit or cost, and it is more sensitive to the circumstances and preferences of the patients than either the opportunity cost method or the replacement cost method [35]. The CVM is more comprehensive than estimations of disease cost that are made using medical costs obtained directly from insurance agencies. The method is referred to as a “contingent” valuation method because it attempts to understand how individuals would act if they were placed in a specific possible and imaginable situation. Similar studies applied to hypothetical conditions have been conducted, including studies on air quality, the preservation of wildlife, the value of programs designed to reduce the risks of respiratory diseases, and the WTP for cures for various diseases [36,37]. Please refer to the previous literature for more details [38,39]. The software employed for data analysis was STATA 12.0. 

### 2.7. Ethics Approval

All study participants provided written informed consent. The study was approved by the Institutional Review Board of the Tzu-Chi Hospital for ethics evaluation with the Ethic Approval Number IRB099-88. 

### 2.8. Implementation

The survey was administered in 15–20 min in-person interviews in private offices at the hospitals. The survey was self-administered, but the interviewers were in attendance to ensure full understanding of the questions by the participants.

## 3. Results 

### 3.1. Patient Background 

Two hundred forty-eight patients were initially invited to be interviewed, and one hundred forty-two patients completed the survey, yielding a response rate of 0.57. A statistical summary of the survey results for the whole sample and by severity grade is presented in Table 1. For the entire sample, the average age (±SD) of the subjects was 73.11 (±9.90) years, and 75% of the subjects were married. Of all of the subjects, 63.11% were past cigarette smokers, 13.93% were current smokers, and 22.95% had never smoked. As the disease severity increased, the proportion of subjects who had never smoked decreased. The average HRQL of the subjects was high when measured using the EQ-5D; an EQ-5D value of 0.84 (±0.21) was obtained on a scale of 0 to 1, with 1 representing perfect health. From the SF-36 measurement, the mental health component summary (MCS) also reflected a close-to-average result of 49.48, where 50 indicates the average health condition of the general population. However, the physical component summary (PCS) value of 37.47 indicated a lower-than-average health condition, suggesting that COPD patents may have less physical strength than the average person.

### 3.2. Comorbidity Background

The subjects’ comorbidity conditions by disease severity grade are presented in Table 2. The most commonly observed comorbidity for COPD patients was hypertension, which affected 31.25% of the subjects; this prevalence was significantly higher than the population mean prevalence in Taiwan. Other comorbidities that had higher incidence rates in the subjects than in the general population included cardiovascular diseases and cancer, with comorbidity rates of 19.51% and 8.93%, respectively. 

### 3.3. Scenario Validity

To analyze patient understanding of the meaning of WTP and whether they found the hypothetical scenario compelling, differences in the respondents’ ratings of the acceptability of the scenario according to their disease stages were examined. The results are presented in Table 3. Patients with the mildest disease stage had the lowest acceptance rate (0.41), and those with more severe disease stages tended to be more accepting of the hypothetical scenario. The results suggest that individuals who suffered more in general appeared to have a greater desire for the cure brought by the magic pill than did individuals who suffered less, which implies that the scenario was clearly understood by the patients.

### 3.4. WTP

The results of WTP for a COPD cure are presented in the left-hand columns of Table 4. The percentage of “yes” responses (as shown in parentheses in the “Count of Yes” column) did not monotonically decrease as the initial bid increased, which is inconsistent with the concept of demand theory. Thus, pooling the violating results backwards with the previous bids, as suggested by the Turnbull distribution, was necessary, and the probability mass point estimates are reported in the right-hand columns of Table 4. With this treatment, the monotonicity assumption for a standard distribution function of the WTP response can be ensured [45]. 

To understand patient WTP and its determinants for a cure for COPD, regression analyses using the maximum likelihood method for closed-ended double-bounded dichotomous questions were performed; the results are presented in Table 5. Income was initially collected as ordinal data, but it was treated as a continuous variable measured at the midpoint of each interval. Only the adjusted WTP bids suggested by the Turnbull distribution model, as presented in Table 4, were estimated. Because collinearity problems among the independent variables can be a problem when different QOL measures, severity grades, and CAT values are under consideration, eight different regression models were tested for robustness by including different categories of variables at a time. The comorbidity variables were coded as dummy variables separately in the models, with 1 indicating the presence of a comorbidity and 0 otherwise. The severity grades and the EQ-5D index were also coded as dummy variables. Although the EQ-5D is a continuous index, it typically exhibits a non-linear impact on WTP. Therefore, separating the EQ-5D index into several categories may better reflect its effect [46]. 

The baseline model, which is presented in column (1) of Table 5, includes only patient background and comorbidities as regressors. This model indicates that age exhibits a polynomial relationship up to the third power and that comorbidities, including diabetes, cancer, and rhinitis, have a positive impact on WTP, whereas metabolic disorders have a negative impact. All results were significant at the 5% significance level. Other models that added the CAT, spirometric severity grade, or various QOL values are reported in columns (2) to (7). Model (8) includes all of the possible control variables in the regression. The regression results indicate that income positively affects the subjects’ WTP although the effect was not significant in all models. The impacts of comorbidities in these additional models were similar to those in the baseline model: diabetes, rhinitis, and cancer exhibited significant positive relationships with WTP in most of the models. With these comorbidities, patient WTP increased by 0.73%–1.11%, 1.33%–1.72%, and 0.71%–1.10%, respectively. The patients with metabolic disorders tended to have a lower WTP by approximately 0.77%–1.39%. Hypertension and cardiovascular disorders, however, did not significantly impact WTP in most of the models. 

Regarding the severity level, both the CAT and spirometric classifications were employed. The former classification emphasizes the disease symptoms of the patients, and the latter is limited to the airflow of the patients’ pulmonary system. Because the two measures can be highly correlated, some of the regression models included only one of the two measures to avoid collinearity problems. The results in Table 5 demonstrate that the CAT had a significant positive impact on WTP, as shown in columns (6) and (8): with a one point increase in the CAT, WTP increased by 0.05%–0.07%, holding all other variables constant. For the spirometric measure of severity, the subjects who were classified as very severe had a significantly higher WTP than did those patients classified as mild (the reference category for comparison). For HRQL, both PCS in the SF-36 and physical health in the WHOQOL exhibited positive relationships with WTP; the results were significant at the 5% level (refer to columns (4) and (5) for the SF-36 and columns (6) and (8) for the WHOQOL). Regarding the EQ-5D index, the results suggest that subjects with an EQ-5D index between 0.5 and 0.8 had a WTP that was 1.16%–1.98% lower than that of subjects with an EQ-5D index less than 0.5. In conclusion, the results indicate that the more severely ill patients and those with lower HRQL scores tended to have higher values of WTP for a COPD cure.

###  3.5. Simulation of WTP for Different Target Groups

After the regression models were constructed, simulations of subject WTP for a complete cure for COPD were performed, and the results are presented in Table 6. Model (8) was used for this purpose because it included the most complete set of regressors and because most of them exhibited significant results. The average WTP for the entire sample was $42,662.37 NT (or $1422.08 USD) per year. WTP increased with income and decreased with age level. The patients of the more severe level and those with the lowest QOL scores tended to have the highest WTP amount. However, the second highest WTP amount was identified for the subjects with the lowest severity grade and the highest QOL. Current smokers were willing to pay more for a cure for COPD than were either former smokers or those whose had never smoked.

## 4. Limitations

Compared with the size of the COPD population in Taiwan, the number of surveyed subjects in our study was small; therefore, a potential selection bias may be present. However, COPD is highly correlated with smoking behavior, and the ratio of male-to-female smokers is 10.9:1 in adults [47]. In our study, most subjects were current or former male smokers, which is consistent with Taiwan’s smoking population profile. In addition, the prevalence of cigarette smoking is generally higher in eastern Taiwan than in western Taiwan [48]. Thus, our sample represented a group of patients who were associated with cigarette smoking. In this regard, the sample population is representative of general COPD patient statistics. Based on the subject distribution across severity level as presented in Table 3, our sample potentially consists of a greater proportion of patients with more severe cases of COPD than the composition of patients in Western countries. Compared to the distribution in the Netherlands [49], in which 28% of COPD cases are mild, 54% are moderate, 15% are severe and 3% are very severe, our percentages of severe and very severe cases are higher and the percentage of moderate cases is lower. This result suggests the possibilities that COPD is not a well-understood disease for the general Taiwanese population and that people with COPD tend not to seek medical help until their conditions develop. The selected hospitals in this study are prestigious hospitals in northern and eastern Taiwan, and each hospital provides services to nearly one thousand COPD patients annually. Thus, the patients referred by their physicians within this time period can be considered a representative sample of the larger population, except that the 0.57 response rate could lead to some degree of selection bias. Another limitation of the study is that the comorbidities of the subjects were self-reported. The subjects are in general not aware of the detailed disease classification. Thus, only broad categories of comorbidities were included in the survey. 

## 5. Discussion and Conclusions

The subjects’ annual average WTP for a complete cure for COPD was $42,662.37 NT (or $1422.08 USD), which is on the lower side of the COPD per capita spending in the U.S [8]. However, it is far exceeds the amount that the Taiwan National Bureau of Health Insurance (NBHI) spends for each patient [17]. The surveyed patients were recruited from outpatient centers, and many end-staged severely ill patients were excluded. Thus, it was not unexpected that the estimated WTP in this research was not particularly high. Nonetheless, the annual WTP of the younger group of patients (aged 55 years or younger) was three times higher than the average WTP, which was equivalent to one-third of the average annual personal income of the same year (approximate $19,034 USD) or quadruple the amount of the Taiwan NBHI spending on each COPD patient. These findings imply the patients’ health burden severely hindered their work performance and may have resulted in productivity loss. 

The regression results consistently indicated that HRQL significantly influenced the patients’ WTP for a COPD cure. Regardless of which HRQL measures were employed, a lower HRQL was associated with a higher WTP. The subjects with more severe symptoms also tended to have a higher WTP for a COPD cure regardless of the CAT or spirometric severity grade measures considered. However, the simulation results reported in Table 6 presented an M-shaped distribution: subjects in the highest and lowest QOL categories or severity grades were willing to pay the highest and second highest amounts of money for a disease cure, respectively. This finding suggests that subjects in the early mild stage of the disease are as concerned about their health condition as are individuals in the most severe stage, which suggests that patients in the initial stages of COPD may find the symptoms alarming and bothersome. This finding may also have an age component: patients at the mild disease stage are typically younger and have more life years ahead of them than patients at other stages, which could explain the higher WTP.

The comorbidities that positively affected patient WTP included diabetes, cancer, hypertension, and rhinitis. The former three comorbidities had higher incidence rates in COPD patients than are reported for the general population. However, the COPD patients with rhinitis had the highest WTP amount relative to the other comorbidities. Rhinitis is considered to be indicative of asthma COPD overlap syndrome (ACOS) [50]; such patients typically have more severe cases of exacerbations and hospitalizations [51]. It is therefore not unexpected that the subjects comorbid with rhinitis had a greater desire for a disease cure. Because tobacco use is known to be highly related to rhinitis [52], this result reinforces the finding that current cigarette smokers are willing to pay the highest amounts for a COPD cure compared with former smokers and nonsmokers. This finding could reflect the psychological desire for redemption. Together, the results suggest that more aggressive effort is necessary to reduce the population’s use of tobacco. 

In conclusion, the results of this study provide directions for the relevant authorities regarding the alleviation of suffering because of COPD. In particular, patients who are younger, current smokers, and comorbid with rhinitis and cancer are those who bear the greatest burden and thus require special attention. Appropriate health promotion measures, medical attention, and early diagnosis and active treatment may be necessary to contain the escalating costs related to COPD and to prevent this epidemic from worsening. 

## Figures and Tables

**Table 1:** Statistical summary of patient backgrounds ^a^.

Severity Variables	Whole	Mild	Moderate	Severe	Very Severe
Observation	142	40	53	30	19
Background					
Age	73.11 (9.9)	75.94 (9.54)	71.11 (9.78)	74.88 (9.72)	69.00 (9.96)
Male	86%	82%	80%	96%	93%
Married	75%	82.85%	64.44%	76.92%	85.71%
Income (×1000 NTD)	256.23 (457.66)	168.0 (262.66)	284.35 (440.62)	175.38 (217.94)	552.8 (932.54)
Cigarette ^b^	25.72 (12.88)	22.5 (13.02)	25.96 (12.63)	27.39 (13.40)	27.12 (12.94)
Height (cm)	161.33 (7.7)	160.34 (7.42)	160.38 (8.52)	162.41 (6.62)	163.96 (6.87)
Weight (kg)	61.58 (14.59)	59.47 (11.2)	62.73 (16.79)	59.26 (11.29)	67.56 (18.11)
BMI	23.62 (5.00)	23.02 (4.02)	24.21 (5.40)	22.70 (4.38)	25.05 (6.28)
Smoking History					
Never	22.95%	31.43	23.91	19.23	0.00
Past	63.11%	54.29	52.17	80.77	92.86
Current	13.93%	14.29	23.91	0.00	7.14
WHO					
Physical	57.82 (18.24)	62.09 (17.55)	57.67 (15.99)	55.77 (18.85)	49.79 (23.57)
Psychological	56.03 (17.40)	60.6 (16.45)	57.33 (15.64)	52.69 (18.73)	45.64 (19.37)
Social	61.52 (17.98)	68.8 (15.39)	62.83 (19.15)	54.58 (16.10)	50.93 (15.57)
Environment	61.08 (15.01)	65.51 (12.09)	61.41 (14.85)	59.04 (16.59)	51.71 (15.79)
EQ-5D	0.84 (0.21)	0.88 (0.20)	0.89 (0.16)	0.79 (0.20)	0.65 (0.32)
SF-36					
PCS	37.47 (12.39)	42.19 (12.66)	37.88 (12.08)	32.30 (10.43)	31.87 (11.04)
MCS	49.48 (11.63)	53.95 (9.75)	47.67 (11.85)	48.98 (12.06)	44.03 (11.97)
Current Health ^c^	71.45 (18.4)	70.48 (13.87)	69.58 (25.17)	72.86 (14.54)	74.23 (16.44)
FEV1% Predicted	54.33 (22.02)	95.72 (14.25)	62.29 (8.8)	39.23 (5.46)	23.99 (3.79)
CAT	15.95 (9.97)	12.06 (9.16)	14.53 (9.47)	20.35 (8.67)	23.14 (9.57)

Note: ^a^ The mean or percentages are reported, and the standard deviations are in parentheses. ^b^ Number of cigarettes smoked per day. ^c^ Self-reported current health status on a 1–100 scale. Abbreviations: NTD: New Taiwan Dollars; BMI, Body Mass Index; PCS, physical component summary; MCS, mental component summary; FEV, forced expiratory volume; CAT, COPD Assessment Test.

**Table 2:** Statistical summary of comorbidity incidence rates.

Severity Comorbidities ^a^	Whole Sample	Mild	Moderate	Severe	Very Severe	Population Incidence ^b^ (%)
Observation	142	40	53	30	19	
(%)						
Rhinitis	4.46	10.00	4.87	0.00	0.00	15–20 [40]
Diabetes	12.5	16.67	9.75	15.38	7.14	12.8 [41]
Hypertension	31.25	30.00	26.82	38.46	35.71	23.30 [42]
Cardiovascular	19.51	7.88	43.47	61.54	64.28	6.8 [42]
Renal	0.61	0.00	4.34	0.00	0.00	2.9 [42]
Liver	1.22	0.83	2.17	0.00	7.14	6.1 [42]
GI	2.74	0.83	0.00	15.38	21.43	6.6 [42]
Blood Abnormal	0.30	0.00	2.17	0.00	0.00	
Neurological	0.61	0.04	2.17	0.00	0.00	
Metabolism	5.79	1.66	15.21	23.07	14.28	10–15 [43]
Cancer	8.93	3.33	9.75	15.38	7.14	0.38 [44]

Note: ^a^ The comorbidity records were self-reported; therefore, only general classifications of comorbidities were provided. ^b^ Selected comorbidity incidence rates based on the availability for the general population in Taiwan are presented in the last column for comparison purposes.

**Table 3:** WTP acceptance rating by disease stage.

Disease Grade	Obs	(%)	Acceptance Rating	(SD)
Mild	40	28.16	0.47	0.50
Moderate	53	37.32	0.54	0.51
Severe	30	21.13	0.78	0.50
Very Severe	19	13.38	0.53	0.50

Abbreviations: WTP: willingness to pay; Obs, observation number; SD, standard deviation.

**Table 4:** Response ratings across the 1st and 2nd bids.

Unrestricted			Turnbull Estimate			
Initial Bids ($ NT)	Total (%)	Count of Yes ^a^ (%)		Total (%)	Count of Yes (%)	Change of Yes
500	18 (12.6)	14 (77.7)	500	18 (12.6)	14 (78.0)	--
1500	24 (15.5)	18 (75.0)	1500	51 (35.9)	24 (47.1)	−30.9%
2000	12 (7.8)	3 (25.0)				
3000	15 (9.7)	3 (20.0)				
4000	19 (12.3)	6 (31.6)	4000	39 (27.5)	18 (46.0)	−1.1%
6000	20 (12.9)	12 (60.0)				
8000	16 (10.3)	13 (81.3)	8000	34 (23.9)	13 (38.2)	−7.8%
10,000	18 (11.7)	0 (0.0)				
Total	142 (100)	69 (48.6)	Total	142 (100.0)	69 (48.6)	

Note: The respondents are assigned into groups with different initial bids. “Total” indicates the number of respondents in the corresponding group. ^a^ represents the number of respondents that answered “yes” to the initial bid.

**Table 5:** Regression results for WTP for a COPD Cure.

Variables	(1)	(2)	(3)	(4)	(5)	(6)	(7)	(8)
Income	0.02	0.03	0.03	0.19 *	0.19	0.10 *	0.17 *	0.31 **
	(0.12)	(0.2)	(0.19)	(1.77)	(1.16)	(1.71)	(1.77)	(1.91)
Age	−3.63 **	−5.67 ***	−5.35 ***	−5.80 ***	−5.84 ***	−4.87 ***	−5.80 ***	−4.61 ***
	(2.21)	(3.32)	(3.14)	(3.34)	(3.43)	(2.99)	(3.47)	(2.78)
Age^2^	0.05 **	0.08 ***	0.08 ***	0.08 ***	0.08 ***	0.07 ***	0.08 ***	0.07 ***
	(2.12)	(3.31)	(3.13)	(3.34)	(3.44)	(3.00)	(3.49)	(2.84)
Age^3^	0.00 **	0.00 ***	0.00 ***	0.00 ***	0.00 ***	0.00 ***	0.00 ***	0.00 ***
	(2.04)	(3.3)	(3.11)	(3.34)	(3.45)	(3.01)	(3.51)	(2.89)
Male	0.08	0.05	0.07	−0.56	−0.52	−0.28	−0.42	−0.72
	(0.19)	(0.09)	(0.14)	(1.1)	(1.01)	(0.6)	(0.83)	(1.55)
Married	0.12	0.47	0.43	0.4	0.57 *	0.17	0.62 *	0.33
	(0.39)	(1.5)	(1.37)	(1.26)	(1.76)	(0.57)	(1.97)	(1.02)
Diabetes	0.73 *	0.81 *	0.77 *	0.90 *	0.94 *	0.68	0.97 **	1.11 **
	(1.73)	(1.71)	(1.63)	(1.81)	(1.87)	(1.52)	(2.07)	(2.25)
Hypertension	0.39	0.57 **	0.51 *	0.59 **	0.35	0.64 **	0.21	0.36
	(1.4)	(2.00)	(1.75)	(1.94)	(1.11)	(2.29)	(0.71)	(1.16)
Cancer	0.97 **	0.76*	0.71 *	1.27 ***	1.29 ***	0.99 **	1.10 **	1.42 ***
	(2.48)	(1.78)	(1.67)	(2.74)	(2.84)	(2.39)	(2.57)	(3.22)
Rhinitis	1.72 ***	1.63 **	1.48 **	1.49 **	1.33 **	1.54 ***	1.43 **	1.54 **
	(2.92)	(2.73)	(2.44)	(2.5)	(2.29)	(2.63)	(2.46)	(2.54)
Cardiovascular	−0.32	−0.46 *	−0.33	−0.31	−0.45 *	−0.32	−0.53 **	−0.52 **
	−1.24)	(1.71)	(1.12)	(1.16)	(1.66)	(1.14)	(1.82)	(1.91)
Metabolic	−0.77 **	−1.12 **	−1.02 **	−1.39 ***	−1.31 ***	−0.93 **	−1.05	−1.12 ***
	(2.09)	(2.71)	(2.46)	(3.15)	(2.98)	(2.37)	(2.53)	(2.64)
BMI		−0.03	−0.03	−0.02	−0.03	0	−0.05	−0.02
		(1.02)	(1.1)	(0.57)	(1.1)	(0.08)	(1.83)	(0.65)
CAT			−0.01			0.05 **	0	0.07 ***
			(0.84)			(2.28)	(0.22)	(3.15)
Moderate				0.05	−0.03		−0.18	−0.13
				(0.13)	(0.08)		(0.48)	(0.34)
Severe				0.42	0.45		0.15	0.19
				(1.07)	(1.16)		(0.41)	(0.46)
Very Severe				1.51 ***	1.35 ***		1.00 **	1.02 **
				(2.75)	(2.47)		(1.89)	(1.87)
PCS				0.04 ***	0.03 **			0.02
				(2.89)	(1.93)			(0.9)
MCS				0.01	0.01			−0.02
				(1.2)	(0.73)			(1.2)
0.8 > EQ-5D > 0.5					−1.16 **		−1.21 **	−1.98 ***
					(2.05)		(2.15)	(3.15)
EQ-5D > 0.8					−0.29		0.19	−0.91
					(0.5)		(0.36)	(1.45)
Physical						0.03 **		0.05 ***
						(2.26)		(2.99)
Psychological						0.03 **		0.02
						(1.97)		(1.07)
Social						0		0.01
						(0.02)		(0.68)
Environment						-0.01		−0.01
						(0.59)		(0.51)
Constant	95.26 **	139.41 ***	132.48 ***	139.15 ***	140.63 ***	116.41 ***	141.24 ***	108.19 ***
	(2.49)	(3.51)	(3.35)	(3.44)	(3.54)	(3.06)	(3.65)	(2.8)

Notes: 1%, 5%, and 10% levels of significance are denoted by ***, **, and *, respectively. *t*-values are in parentheses. WTP and income were measured using a natural logarithm. The comorbidities were coded as dummy variables. The omitted dummy reference variable levels were the mild severity level and the EQ-5D < 0.5 category. Age is measured in polynomial form: Age ^2^ denotes age square and Age ^3^ denotes age cube.

**Table 6:** Simulated WTP for subjects in different target groups.

Categories	Obs. Number	Computed WTP (NTD)	SE
Entire Sample	142	42,662.37	79,054.74
Income (monthly, NTD)			
<20,000	85	27,211.16	33,227.24
20,000–49,999	35	51,534.84	80,865.79
>50,000	22	64,441.95	122,395.90
Age			
<55	8	171,272.04	145,407.36
55–65	50	45,431.16	89,536.68
65–75	64	32,450.64	34,407.96
75–85	76	49,335.36	17,867.04
>85	26	12,169.44	11,482.92
Severity Grade			
Mild	40	44,731.92	72,303.92
Moderate	52	24,326.14	26,752.69
Severe	32	25,241.34	19,599.26
Very severe	18	125,524.62	169,071.01
Comorbidities			
Diabetes	18	61,073.70	88,790.42
Hypertension	44	58,604.55	85,957.99
Rhinitis	9	132,621.12	112,696.32
Cancer	13	80,836.66	47,989.64
Cardiovascular	28	44,001.67	75,540.11
Metabolic Disorder	13	12,523.60	15,835.73
EQ-5D			
Index < 0.5	12	60,364.28	91,470.65
0.5–0.8	32	11,127.33	9067.98
>0.8	98	40,426.63	64,340.33
CAT			
<10	48	58,271.61	88,496.54
10–20	48	27,128.29	27,106.44
20–30	30	22,499.16	21,558.13
30–40	16	84,327.10	162,671.98
Smoking History			
Never	32	28,211.17	33,227.25
Former	90	41,962.16	71,578.79
Current	20	71,398.22	122,523.80

Note: Obs: observation number; CAT: COPD assessment test; SE: standard error.
